# A Young Female Case of Polyarteritis Nodosa Presenting With Multisystem Involvement and Acute Abdomen: A Case Report

**DOI:** 10.7759/cureus.20778

**Published:** 2021-12-28

**Authors:** Rahul Navab, Visweswara Reddy Yeragudi Jangamareddy, Nagabushana V Midthala, Thinagaran Kamalakannan

**Affiliations:** 1 Internal Medicine, People’s Education Society Institute of Medical Sciences and Research, Kuppam, IND; 2 Internal Medicine, Narayana Medical College and Hospital, Nellore, IND; 3 General Surgery, Shri Sathya Sai Medical College and Research Institute, Ammapettai, IND

**Keywords:** necrotizing inflammation, explorative laparotomy, ascites, leukocytoclastic vasculitis (lcv), hepatitis b virus (hbv)

## Abstract

Polyarteritis nodosa (PAN) is a medium vessel vasculitis that affects multiple organ systems except the lungs. It is transmural segmental necrotizing inflammation associated with fibrinoid necrosis. Hepatitis B virus (HBV) infection is strongly associated with PAN. It commonly affects medium-sized muscular arteries and typically involves renal, coronary, and mesenteric vessels, but not pulmonary arteries. Vascular lesions occur mostly at bifurcations in medium-sized muscular arteries. A case of polyarteritis nodosa was reported in a 21-year-old female who presented with blackish discoloration of feet, loss of appetite, loss of weight, colicky pain, and distension of the abdomen. Leukocytoclastic vasculitis was confirmed by skin biopsy; ascites was detected on computed tomography and chest and abdomen (erect) radiograph; and explorative laparotomy was done, but she died on the second postoperative day due to septicemic shock and acute renal and respiratory failure.

## Introduction

Vasculitis is an inflammation and weakening of blood vessel walls, causing damage to the skin, kidney, gastrointestinal tract, brain, heart, peripheral, and central nervous system. Polyarteritis nodosa (PAN) is a classical collagen disease with a poor prognosis that demonstrates systemic necrotizing vasculitis of small and medium-sized arteries [1]. PAN has a peak incidence between the ages of 40 and 50, with a male to female ratio of 2:1. The common presentation of PAN includes fever, body ache, joint pains, and loss of weight in association with multisystem disease features [2]. Clinical manifestations are mainly due to ischemia and infarction of affected tissues and organs. Gut involvement is found in 14% to 65% of cases of polyarteritis nodosa, and abdominal pain after meals due to gut ischemia is a frequent symptom [3]. Bowel perforation, cholecystitis, gallbladder infarctions, and acute pancreatitis are the common causes of an acute abdomen in polyarteritis nodosa. PAN often presents early and frequently with peripheral neuropathy in 50% to 75% of cases; however, the central nervous system is affected in 2% to 10% of cases in an advanced course of disease [4].

## Case presentation

A 21-year-old female presented with blackish discoloration of feet, loss of appetite, weight loss of 5 kg in two months duration with a 15-day history of low-grade fever, colicky pain, and distension of abdomen. She had a two-year history of burning sensation of feet and joint pains, and she was on long-term steroids which were discontinued two months ago in view of abdominal pain.

**Figure 1:**
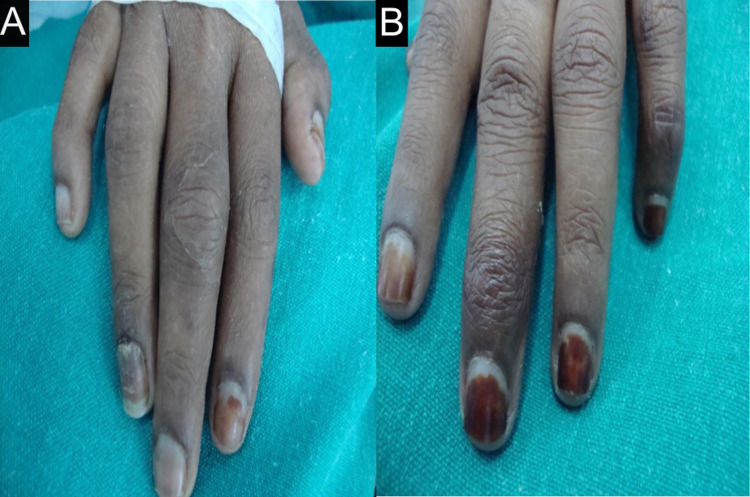
Swelling of small joints Asymmetric swelling of small joints on (A) right and (B) left hands.

**Figure 2:**
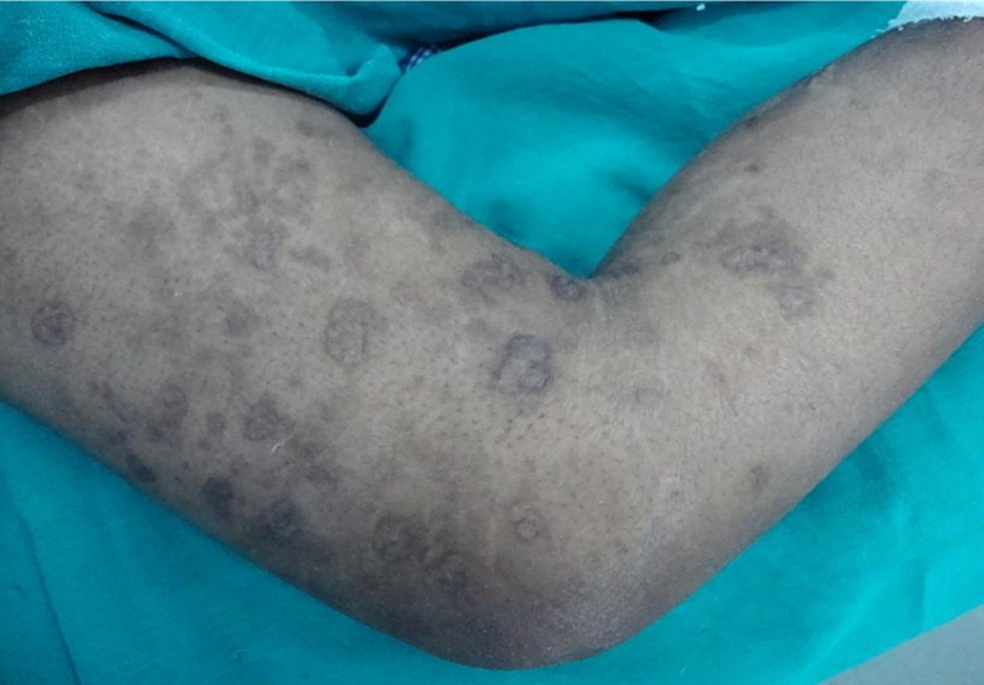
Healed scars on the right thigh

**Figure 3:**
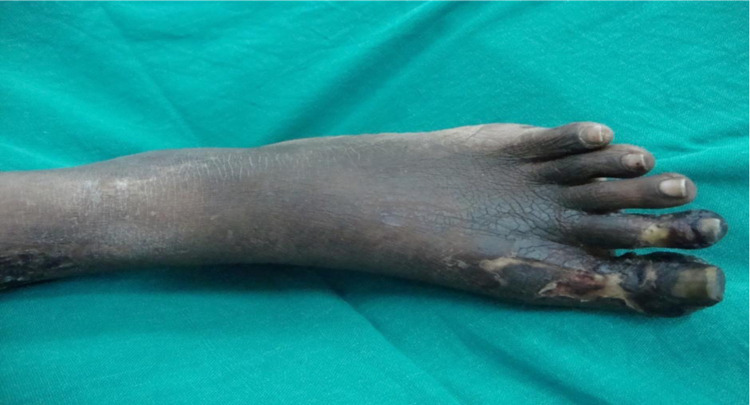
Gangrene of feet with hyperpigmentation

**Figure 4:**
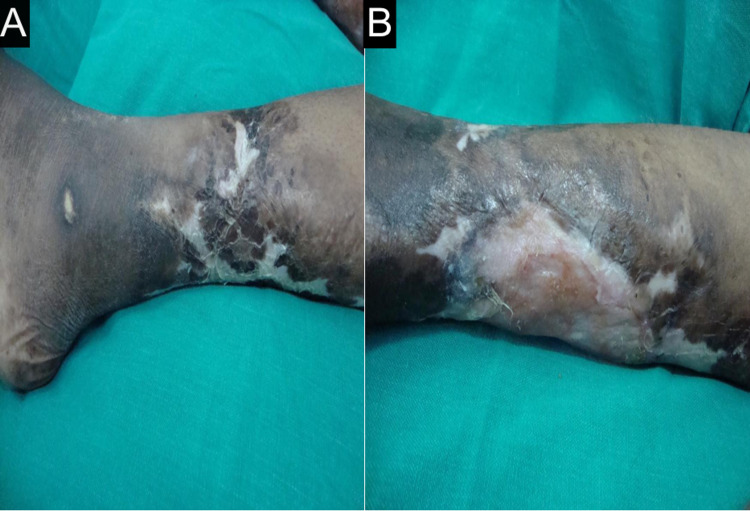
Healed ulcers Healed ulcers with a scar formation on (A) right and (B) left legs.

She was a known case of hypertension but was not a diabetic. On examination, the patient was of average build and looked ill. Pallor and icterus were present. Heart rate (HR) was 94 beats per min (bpm), regular in rhythm. Blood pressure was 160/100 mmHg. Dorsalis pedis and anterior and posterior tibial arteries were feeble on the left side. Asymmetric swelling of small joints of hands (Figures 1A, 1B) with discoloration of overlying skin, healed scars on the thigh (Figure 2), gangrene of feet (Figure 3), and healed ulcers with a scar formation on right and left legs (Figures 4A, 4B) were noted. Hyperesthesia over both the feet was present. Mild nontender hepatomegaly and free fluid were present.

**Figure 5:**
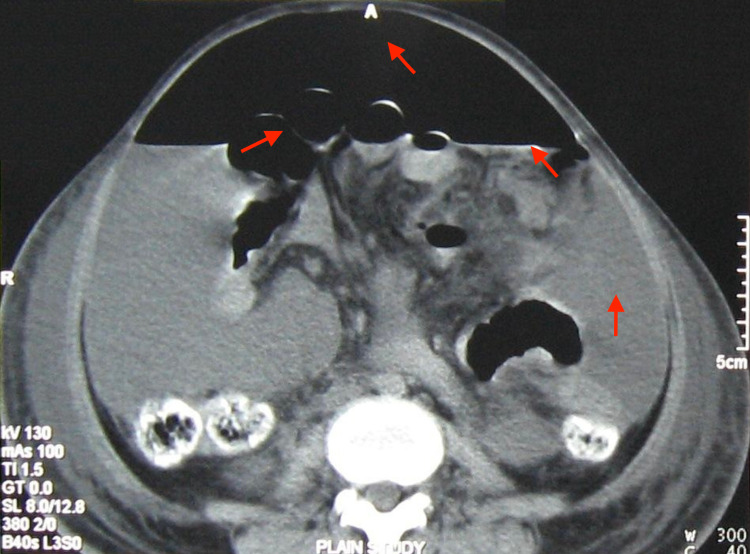
Computed tomography axial section reveals free intraperitoneal air in the anterior abdomen, diffuse ascites with air-fluid levels, and a few gas-filled small bowel loops (red arrows) TI: Time per rotation, GT: gantry tilt, A: anterior, R: right, C: center, W: window, SL: slice level.

**Figure 6:**
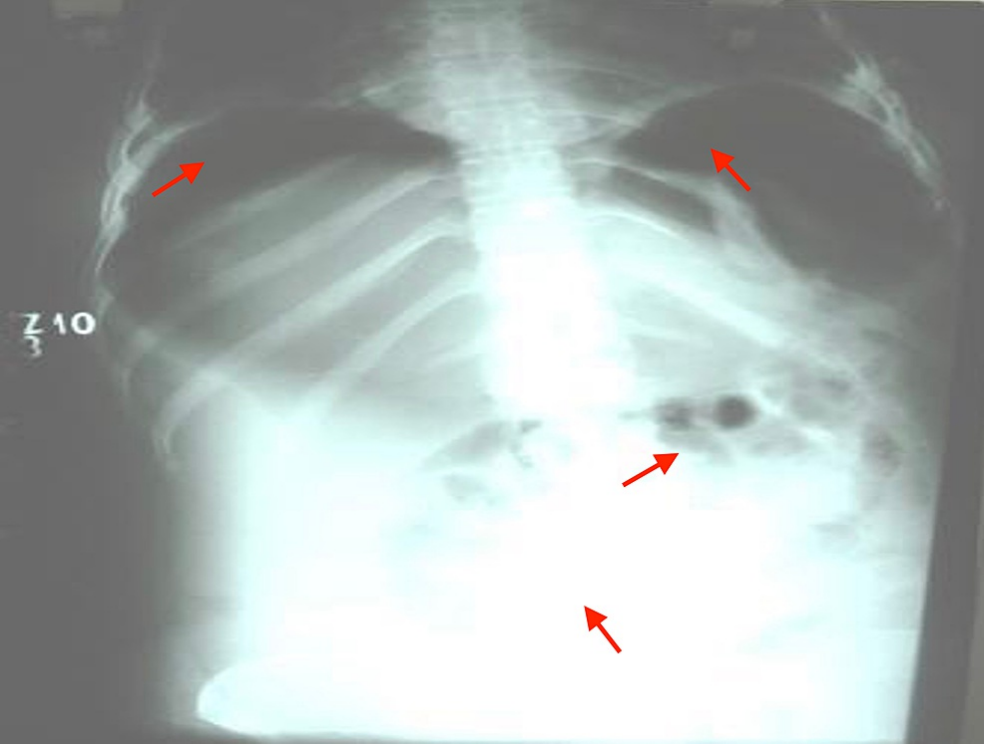
Chest and abdomen (erect) radiograph reveals air under diaphragms, diffuse opacity in the abdomen, and a few gas-filled bowel loops in the abdomen (red arrows)

Her investigations revealed microcytic hypochromic anemia (hemoglobin of 7.5 g/dl) with erythrocyte sedimentation rate (ESR) of 70 mm/h in the first hour. Her prothrombin time (31 seconds) and activated partial thromboplastin time (aPTT) (three minutes) were prolonged along with an international normalized ratio (INR) of 4.3. High serum bilirubin (3.1 mg/dl) was reported. Skin biopsy report confirmed "leukocytoclastic vasculitis" and perinuclear antineutrophil cytoplasmic antibodies were positive. Initial abdominal ultrasound was normal. Initially, abdominal computed tomography revealed hepatomegaly and bulky pancreas. Urea and creatinine levels were elevated. Arterial Doppler of left dorsalis pedis revealed intermittent flow with loss of normal triphasic flow pattern. Repeat computed tomography (Figure 5) was performed along with a chest and abdomen (erect) radiograph (Figure 6) which revealed the following imaging findings. 

**Figure 7:**
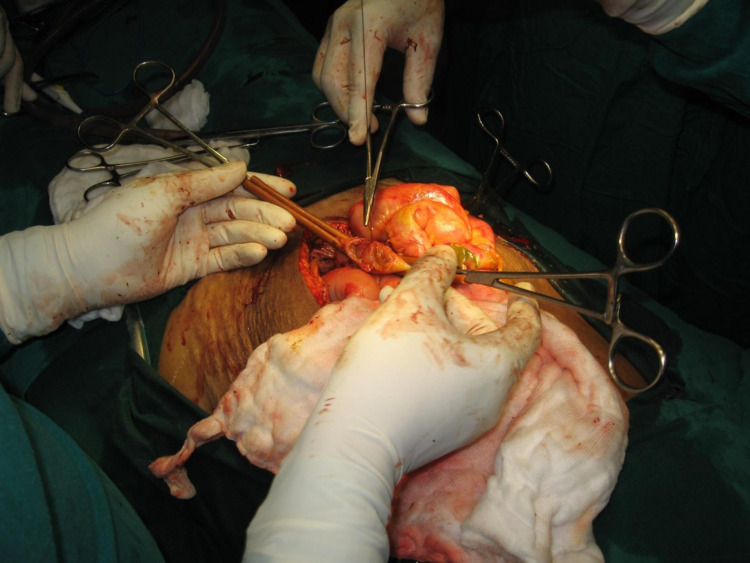
End-to-end anastomosis of bowel

Ascitic fluid analysis revealed an exudate with *Escherichia coli *and *Staphylococcus aureus* on culture and sensitivity. Explorative laparotomy was done on day 15, it revealed 1 l of contaminated feculent peritoneal fluid with three perforations at proximal jejunum, ileojejunal junction, and distal ileum. Edematous and gangrenous bowel in midileum and bulky pancreas and gangrenous and necrotized greater omentum were present. Resection and end-to-end anastomosis of the bowel was performed (Figure 7).

**Figure 8:**
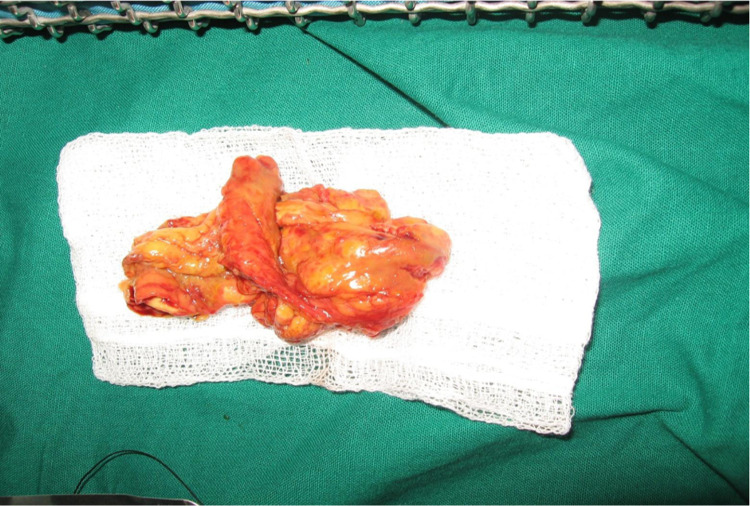
Resected specimen of the bowel

Resected specimen of the bowel is shown in Figure 8.

Our patient had a typical presentation with mild fever, loss of appetite, loss of weight, hypertension, kidney failure, healed ulcers with scar formation, gangrene of feet, abdominal pain as a result of small bowel perforation, and pancreatitis. Based on the above findings, PAN was diagnosed.

She died on the second postoperative day, the cause of death being septicemic shock and acute renal and respiratory failure.

## Discussion

Polyarteritis nodosa (PAN) is a well-recognized but rare cause of vasculitis affecting the peripheral nerves, skin, kidney, and gastrointestinal tract. Its association with the hepatitis B virus has declined from 30% to 7%, thanks to preventive measures [3]. In addition to the systemic idiopathic form, the so-called “idiopathic generalized PAN,” there are 2 clinical variants of this disease viz., “cutaneous PAN” and “hepatitis B virus (HBV)-associated PAN” [5]. Signs and symptoms of PAN are primarily attributable to diffuse vascular inflammation and ischemia of affected organs [6]. The commonest organ manifestation is neurologic with mononeuritis multiplex, followed by skin lesion, abdominal pain from mesenteric ischemia, and renal infarction [7]. In skin, palpable purpura, livedo reticularis, ulceration, and infarction are most common, and pathological changes include necrotizing inflammation and vessel occlusion [2]. Renal hypertension may occur due to multiple renal infarcts.

In any case of acute pain in the abdomen with bilateral pitting edema of the legs and renal dysfunction, the clinical suspicion of PAN should be high and possible occlusive mesenteric vasculitis with or without bowel infarction should be suspected [8]. Gastrointestinal involvement is mainly responsible for morbidity and mortality in patients with PAN. Gut perforation attributable to mesenteric artery occlusion carries a poor outcome [3]. Our patient was diagnosed as PAN with secondary anemia, polyarthritis, gangrene of feet, pancreatitis, ascites, ischemic ileitis with perforations, and peritonitis.

**Table 1:** Criteria for PAN Classification of PAN (polyarteritis nodosa) as per American College of Rheumatology 1990 criteria [9].

PAN Criteria
Weight loss equal to 4 kg or more
Livedo reticularis
Testicular pain or tenderness
Body ache, weakness, or leg tenderness
Mononeuropathy or polyneuropathy
Diastolic blood pressure greater than 90 (mmHg)
Elevated blood urea nitrogen or creatinine
Hepatitis B infection
Arteriogram showing aneurysms or occlusions of the visceral arteries
Biopsy of a small or medium-sized artery containing polymorphonuclear neutrophils

Five of the above-listed criteria were present in our patient. Diagnosis often requires the integration of clinical, angiographic, and biopsy findings [5]. Glomerular renal disease is absent in PAN and should prompt consideration of another diagnosis (microscopic polyangiitis) [8]. Untreated PAN patients die within two years.

Treatment requires high-dose glucocorticoids and immunosuppressants, in addition to supportive treatment. The combined therapy of steroids and cyclophosphamide is better than steroids alone. In non-HBV PAN, steroids and cyclophosphamide are indicated for severe disease. In HBV-related PAN, to diminish the viral load, a two-week course of steroids is started along with plasma exchanges and an antiviral agent, such as interferon alfa to facilitate immune complex [3].

## Conclusions

Systemic vasculitis especially PAN should be thought of in the differential diagnosis of a young patient with features of multisystem involvement. PAN is an uncommon cause of dyspepsia and acute abdomen. Healed scars and gangrene of limbs need a total workup including biopsy to establish the diagnosis of PAN. Pulmonary arteries are not involved generally, for reasons not yet known. PAN continues to be a challenge both in diagnosis and management. Proper management according to the severity of the disease should be given to reduce early mortality from PAN. Relapses may occur if the disease is diagnosed late. The patient should be followed on regular and long-term visits. All the necessary laboratory investigations and imaging tests must be monitored to prevent the further progression of the disease. Early diagnosis and treatment will increase the survival rate. Untreated, the cases almost always end fatally.
